# Integrated alkaline pretreatment and surfactant-assisted hydrolysis for high-yield Manno-oligosaccharides production from spent coffee grounds

**DOI:** 10.1016/j.fochx.2025.102892

**Published:** 2025-08-06

**Authors:** Donglin Xin, Hong Yin, Zhenni Jiao, Ganqiao Ran

**Affiliations:** a Shaanxi Key Laboratory of Qinling Ecological Security, Bio-Agriculture Institute of Shaanxi, Shaanxi Academy of Sciences, Xi'an 710043, Shaanxi, PR China; b Key Laboratory of Biomedical Information Engineering of Ministry of Education, School of Life Science and Technology, Xi'an Jiaotong University, Xi'an 710049, Shaanxi, PR China

**Keywords:** Lignin, Manno-oligosaccharides, Tween 80, Enzymatic hydrolysis

## Abstract

In this study, a process optimization strategy was established to enhance the efficiency of manno-oligosaccharides (MOS) production from spent coffee grounds (SCG), using a sequential approach of aqueous ammonia pretreatment and surfactant-assisted enzymatic hydrolysis. Aqueous ammonia pretreatment (25 % NH₃·H₂O, 70 °C, 48 h) significantly disrupted the lignocellulosic matrix, achieving 67.1 % lignin removal and resulting in more than a threefold increase in MOS yield. The addition of non-ionic surfactants (Tween 80 and PEG 6000) further improved MOS yields by mitigating the non-specific binding of mannanase to lignin. Among them, Tween 80 exhibited superior performance, increasing the MOS yield to 62.3 % at a concentration of 5 mg/mL. This integrated strategy provides a practical and effective means of valorizing SCG into functional oligosaccharides through optimized chemical and enzymatic processes.

## Highlights

• Sequential ammonia pretreatment and hydrolysis produce high-yield MOS from SCG.
• Delignification increased MOS yield by threefold during enzymatic hydrolysis.
• Tween 80 was more effective than PEG 6000 in enhancing MOS production.
• Up to 62.3 % of mannan in pretreated SCG was converted into manno-oligosaccharides.

## 1 Introduction

The global consumption of coffee generates vast quantities of SCG, a lignocellulosic biomass rich in polysaccharides such as cellulose and hemicellulose, particularly mannan (Campos-Vega et al., 2015; Zhao et al., 2024). While typically discarded or used as low-value fuel or compost, SCG represents an underutilized resource with potential for valorization into high-value bioactive compounds. Among these, MOS, derived from the partial hydrolysis of mannan, have attracted considerable interest due to their prebiotic properties, which include promoting beneficial gut microbiota and enhancing immune function (Cho et al., 2022; Im et al., 2025). However, the efficient release of MOS from SCG remains challenging due to the recalcitrant nature of the biomass, especially the presence of lignin, which hinders enzymatic accessibility to mannan (Shaikh-Ibrahim et al., 2025; Zheng et al., 2024).

To overcome the inhibitory effect of lignin on the enzymatic hydrolysis of hemicellulose in lignocellulosic biomass, various pretreatment methods have been developed (Choubane et al., 2025; Fogarin et al., 2025; Huang et al., 2024). Acid pretreatment can dissolve hemicellulose into the hydrolysate, allowing the subsequent enzymatic conversion of solubilized hemicellulose into oligosaccharides. This process helps avoid the inhibitory effects of lignin on hemicellulase activity during oligosaccharide production (de Oliveira Santos et al., 2018; Fogarin et al., 2025). However, acid pretreatment often results in the degradation of target oligosaccharides and the formation of fermentation inhibitors such as furfural and hydroxymethylfurfural (Liu et al., 2022). In contrast, alkaline pretreatment is more effective in selectively removing lignin while better preserving carbohydrate components, thereby enhancing enzymatic accessibility to hemicellulose (Xu et al., 2019; Xu et al., 2020). Among alkaline reagents, aqueous ammonia has emerged as a promising alternative due to its lower corrosiveness, recyclability, and ability to swell biomass, facilitating lignin removal and enhancing enzyme accessibility (Liu et al., 2013; Zhao et al., 2020).

Despite the advantages of aqueous ammonia pretreatment, residual lignin may still adsorb enzymes non-productively, reducing hydrolysis efficiency (Wu et al., 2023; Yuan et al., 2021). Non-ionic surfactants such as Tween 80 and PEG 6000 have been reported to mitigate this issue by blocking lignin binding sites, thereby preserving cellulase activity and improving glucose yield (Lai et al., 2020; Li et al., 2016). However, whether surfactants can enhance the enzymatic hydrolysis efficiency of mannan remains unclear. Moreover, although both aqueous ammonia pretreatment and surfactant-assisted enzymatic hydrolysis have been individually applied to improve the enzymatic digestibility of lignocellulosic biomass, their integrated application for the specific goal of maximizing MOS production from SCG remains unexplored.

In this study, an integrated strategy involving sequential aqueous ammonia pretreatment and surfactant-assisted enzymatic hydrolysis was developed to enhance the production of MOS from SCG. The effects of pretreatment severity on lignin removal and mannan accessibility were systematically evaluated, followed by the application of non-ionic surfactants to mitigate non-productive adsorption of enzymes onto residual lignin. This study presents an efficient approach for the valorization of SCG into functional oligosaccharides, supporting sustainable biomass utilization and the development of high-value prebiotic products.

## 2 Material and methods

### 2.1 Materials

The SCG used in this research were sourced from a local Starbucks store in Xi'an, China. Standards of MOS with DP 2–6 were sourced from Megazyme (Wicklow, Ireland) for use in this study. All chemicals and reagents used throughout the experiments were of analytical grade and purchased from Sigma-Aldrich. The endo-1,4-β-mannanase (MAN) derived from *Aspergillus niger* was purchased from Megazyme (Bray, Wicklow, Ireland) as a liquid enzyme preparation with a protein concentration of 9.8 mg/mL, according to the manufacturer's specifications.

### 2.2 Aqueous ammonia pretreatment

SCG were subjected to pretreatment using 25 wt% aqueous ammonia (analytical grade, purchased from Tianjin Damao Chemical Reagent Factory) at temperatures ranging from 50 to 70 °C for a duration of 24 to 48 h, conducted in screw-capped bottles with a solid-to-liquid ratio of 1:10. Following pretreatment, the SCG were rinsed thoroughly with distilled water until the washings reached a neutral pH. The resulting solid fraction was then air-dried and stored at −20 °C for subsequent compositional analysis and enzymatic hydrolysis experiments.

### 2.3 Enzymatic hydrolysis

Enzymatic hydrolysis of aqueous ammonia-treated SCG was performed using MAN at dosages of 0.05, 0.1, 0.5, 1, and 5 mg protein per gram of dry matter (DM), in a total reaction volume of 1 mL. The reactions were carried out in 50 mM sodium citrate buffer (pH 5.0) at 60 °C for 6, 24, and 48 h. Incubation was conducted in a shaking incubator operating at 200 rpm. At predetermined intervals, aliquots were taken and immediately subjected to boiling for 10 min to halt enzymatic activity. After cooling, the samples were centrifuged at 10,000 ×*g* for 10 min, and the resulting supernatants were collected for analysis of monosaccharides and MOS.

### 2.4 Enhancing MOS yield by surfactants

To evaluate the impact of surfactants on MOS production, Tween 80 or PEG 6000 were incorporated at concentrations ranging from 1 to 5 mg/mL into the enzymatic hydrolysis system. The hydrolysis was performed using MAN (1 mg protein/g DM) on SCG pretreated at 70 °C for 48 h, with a substrate concentration of 5 % (*w*/*v*). Reactions were carried out in 1 mL volumes within 50 mM sodium citrate buffer (pH 5.0), incubated at 50 °C and agitated at 200 rpm for 48 h. Upon completion, the mixtures were centrifuged, and the resulting supernatants were subjected to HPLC analysis for quantification of MOS. The protein content in the supernatant was quantified using a BCA assay kit obtained from Beyotime Biotechnology (PR China).

### 2.5 Carbohydrate analysis

The chemical composition of SCG was determined according to the protocol established by the National Renewable Energy Laboratory (NREL) (Sluiter et al., 2008). Monosaccharides and MOS were analyzed using an Agilent 1260 Infinity II system (Agilent Technologies, USA) equipped with a refractive index detector. For MOS analysis, separation was achieved on a Waters Sugar-Pak I column maintained at 80 °C, using ultrapure water containing 50 mg/L Ca-EDTA as the mobile phase at a flow rate of 0.4 mL/min. Monosaccharide analysis was carried out using a Rezex RPM-Monosaccharide Pb^2+^ column (Phenomenex, USA), with ultrapure water as the mobile phase and a flow rate of 0.6 mL/min. Peak identification and quantification were performed by comparing retention times with those of authenticated standards. The monosaccharides (M1) and MOS yields were determined following the method described by Xin et al. (2024), and unless otherwise specified, were calculated based on the dry weight of the pretreated SCG solids.

## 3 Results and discussion

### 3.1 Chemical composition of SCG

The changes in the contents of cellulose, mannan, and lignin in SCG before and after AA pretreatment were analyzed (Table S1). Previous studies have reported that galactomannan is the predominant polysaccharide in SCG (Simões et al., 2010). Consistent with previous findings, the raw SCG used in this study contained a high level of galactomannan (26.4 %), which exceeded the cellulose content (12.7 %). Notably, approximately 90 % of mannan and over 90 % of cellulose were retained in the solid fraction after aqueous ammonia pretreatment, indicating minimal polysaccharide loss during the process. In addition, the lignin content in raw SCG was 36.6 %. After aqueous ammonia pretreatment, the lignin content was significantly reduced. Specifically, lignin content decreased to 25.7 % after treatment at 50 °C for 24 h, corresponding to a lignin removal rate of 59.1 %. Under more intensive conditions (70 °C, 48 h), the removal rate increased to 67.1 %. These results are in agreement with previous reports demonstrating the effectiveness of aqueous ammonia pretreatment in removing lignin from lignocellulosic materials such as oil palm empty fruit bunches, corn stover, sugarcane bagasse, and bamboo (Jung et al., 2011; Xin et al., 2015; Yu et al., 2013; Zhu et al., 2018). These findings further confirm the high efficiency of lignin removal from SCG by aqueous ammonia pretreatment. The removal of lignin resulted in a marked proportional increase in galactomannan content, reaching approximately 40 % of the residual solids. This high proportion of galactomannan suggests that aqueous ammonia-pretreated SCG is a highly suitable substrate for the production of MOS.

### 3.2 Effect of MAN loading on aqueous ammonia-pretreated SCG hydrolysis

To investigate the effect of MAN loading on the enzymatic production of MOS from aqueous ammonia-pretreated SCG, SCG pretreated at 70 °C for 24 h was used as the substrate, and different doses of MAN were applied to assess their impact on MOS yield (Fig. S1). The results showed that both MOS and monosaccharide yields increased with increasing MAN dosage in the range of 0.05 to 1 mg/g DM. At a MAN loading of 1 mg/g DM, the MOS and monosaccharide yields reached 39.2 % and 12.2 %, respectively. However, when the MAN dosage was further increased to 5 mg/g DM, the MOS yield decreased to 35.2 %, while the monosaccharide yield increased to 32.2 %. This suggests that excessive MAN led to further hydrolysis of MOS into monosaccharides, which is unfavorable for maximizing MOS production. Therefore, a MAN dosage of 1 mg/g DM was selected for subsequent enzymatic hydrolysis experiments.

### 3.3 Effect of pretreatment severity on the enzymatic hydrolysis of mannan in SCG

**Fig. 1:**
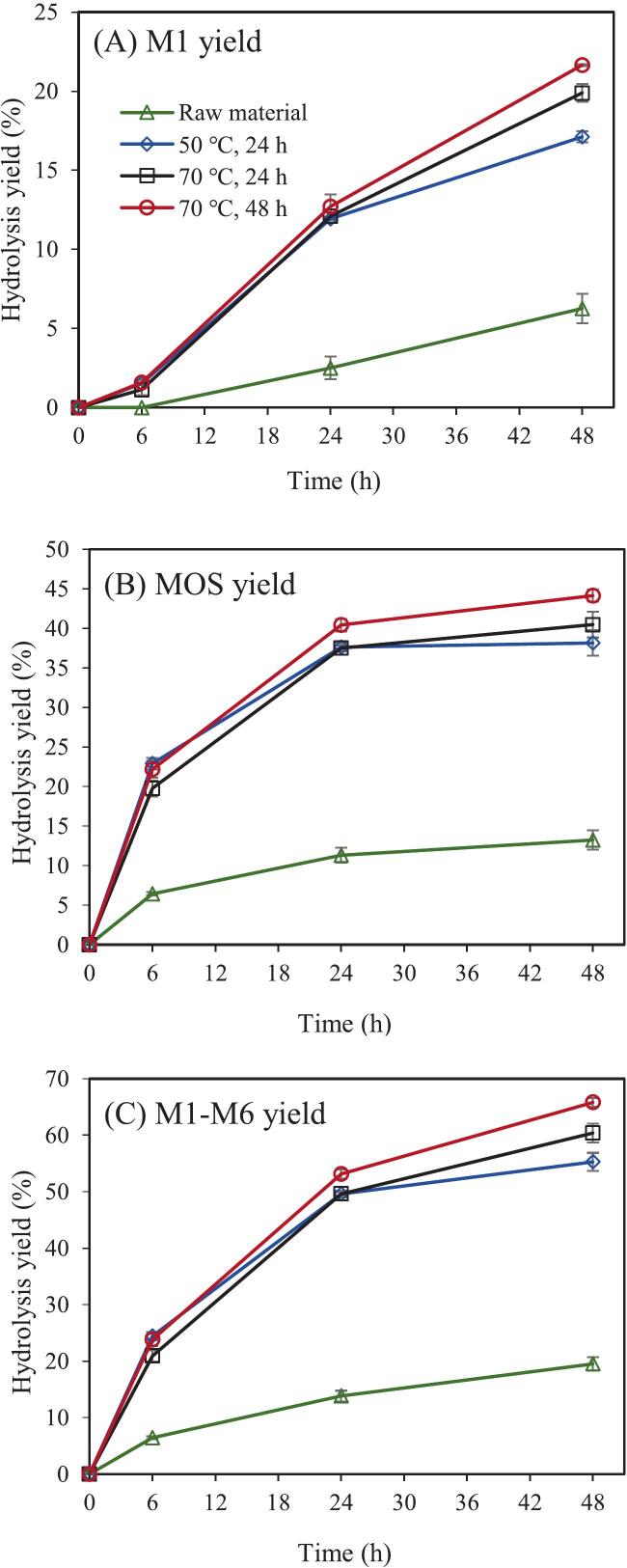
Hydrolysis of aqueous ammonia pretreated spent coffee ground by MAN (1 mg/g DM) at 60 °C and pH 5.0 for 6, 24, and 48 h. The error bars represent the standard errors of three independent experiments.

The degradability of galactomannan in SCG subjected to different aqueous ammonia pretreatment conditions was further evaluated to determine the most suitable condition for MOS production (Fig. 1). Untreated SCG was used as a control, and the hydrolysis yields were calculated based on the dry weight of untreated SCG. As shown, the enzymatic hydrolysis efficiency of untreated SCG was low, with MOS and M1 yields reaching only 13.2 % and 6.3 %, respectively, after 48 h of hydrolysis. The limited hydrolysis efficiency is likely due to the high lignin content in the raw material (36.6 %, Table S1), as previous studies have shown that lignin in lignocellulosic biomass such as SCG is tightly bound to polysaccharides (e.g., galactomannan), thereby restricting enzyme accessibility and significantly hindering the hydrolysis of galactomannan (Gu et al., 2020).

After aqueous ammonia pretreatment, the hydrolysis efficiency of mannan in SCG improved markedly. For SCG pretreated at 50 °C for 24 h, the mannan hydrolysis rate reached 55.3 % (Fig. 1C), with MOS and M1 yields of 38.2 % (Fig. 1B) and 17.1 % (Fig. 1A), respectively. Similar to previously reported mannan hydrolysates from SCG (Magengelele et al., 2023), the main MOS components were mannobiose and mannotriose (Table S2). Previous studies have demonstrated that the prebiotic efficacy of oligosaccharides—particularly their capacity to stimulate the growth and metabolic activity of beneficial gut microorganisms—is closely associated with their degree of polymerization (DP) (Biedrzycka & Bielecka, 2004; Gullón et al., 2011). In particular, oligosaccharides with lower DP values tend to exhibit markedly stronger prebiotic effects compared to those with higher DP (Biedrzycka et al., 2004). In this context, the MOS generated under the present experimental conditions, which are primarily composed of low-DP components, are anticipated to exhibit robust prebiotic potential and offer promising prospects for applications in functional foods and health-related products.

At the highest severity of pretreatment applied in this work (70 °C for 48 h), the mannan degradation rate further increased to 65.8 %, with MOS and M1 yields reaching 44.1 % and 21.7 %, respectively. These results indicate that the degradability of mannan in SCG increases with the severity of aqueous ammonia pretreatment. This phenomenon is likely due to the more extensive removal of lignin under harsher pretreatment conditions, as shown in Table S1. In lignocellulosic biomass, lignin is tightly associated with carbohydrate polymers, which hinders the accessibility of MAN to mannan and reduces the efficiency of enzymatic hydrolysis. The removal of more lignin exposes a greater portion of mannan, facilitating the binding of MAN to its substrate and thereby enhancing the hydrolysis efficiency.

Liu et al. (2013) investigated the effect of aqueous ammonia pretreatment with varying severities on lignin removal and hemicellulose hydrolysis efficiency in miscanthus. They found that the hemicellulose hydrolysis yield increased with the severity of the pretreatment. Under the condition of 30 % aqueous ammonia at 150 °C for 1 h, the lignin removal rate reached approximately 65 %, and the hemicellulose hydrolysis yield of miscanthus increased to 70 %. Similarly, the study by Tsafrakidou et al. (2023) demonstrated that aqueous ammonia pretreatment significantly enhanced the enzymatic hydrolysis of both cellulose and hemicellulose in SCG, and this enhancement was closely associated with lignin removal. These findings are consistent with the conclusions of this study, further suggesting the direct relationship between lignin removal and the enzymatic hydrolysis efficiency of hemicellulose in lignocellulosic biomass.

**Fig. 2:**
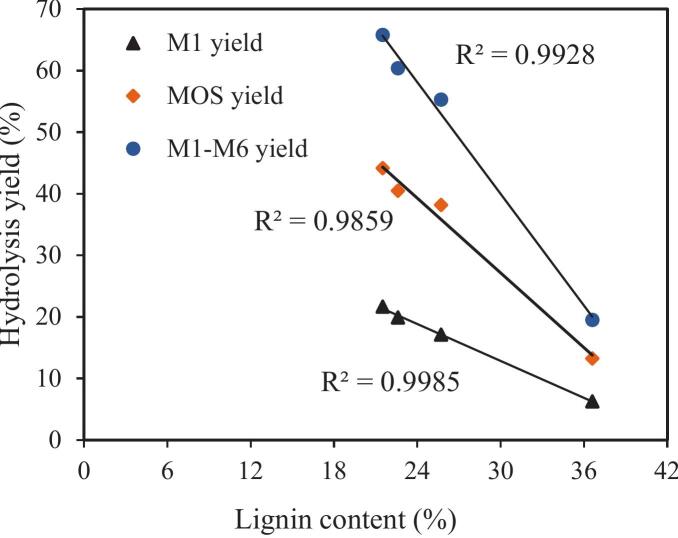
Relationship between lignin content and mannan hydrolysis during the hydrolysis of aqueous ammonia pretreated spent coffee ground by MAN (1 mg/g DM) at 60 °C and pH 5.0 for 48 h.

To further validate this hypothesis, a correlation analysis was conducted using the lignin content data from Table S1 and the mannan degradation yields from Fig. 1 (Fig. 2). The results showed a clear negative correlation between lignin content in SCG and the degradation yield of mannan, including both oligosaccharide and monosaccharide yields. This confirms that lignin in the raw material is one of the key factors limiting the efficient enzymatic hydrolysis of mannan in SCG.

### 3.4 Enhancing MOS yield by surfactants addition

Although aqueous ammonia pretreatment removed a significant portion of lignin, the pretreated SCG still contained approximately 20 % lignin. This residual lignin can continue to negatively impact enzymatic hydrolysis through non-productive adsorption (Rahikainen et al., 2013; Wu et al., 2023). Previous studies have shown that the addition of non-ionic surfactants, such as Tween 80, during lignocellulose hydrolysis can effectively reduce the non-productive binding of lignin to cellulolytic enzymes, thereby improving cellulose saccharification (Li et al., 2016; Wang et al., 2020; Wang et al., 2023). However, whether surfactants can also mitigate the non-productive adsorption of mannanases onto lignin and subsequently improve MOS yields has not yet been reported.

**Fig. 3:**
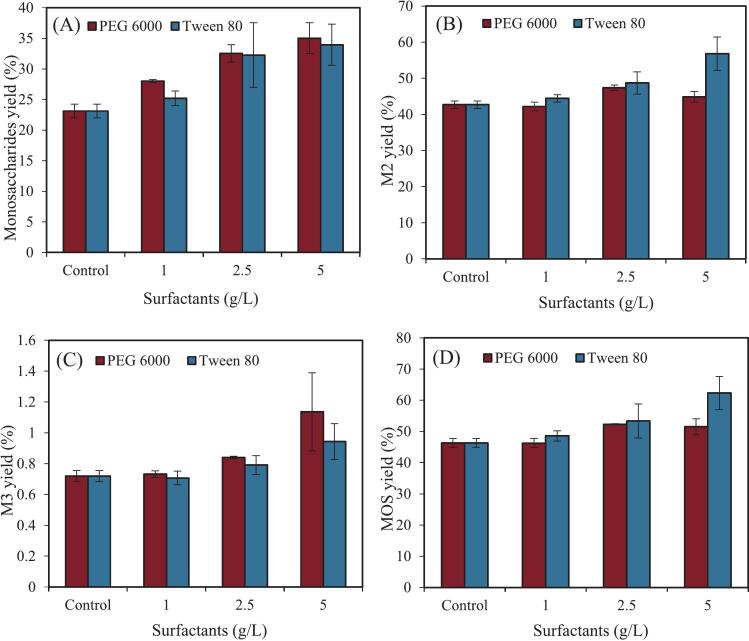
Hydrolysis of aqueous ammonia pretreated spent coffee ground by MAN (1 mg/g DM) and surfactants at 60 °C and pH 5.0 for 48 h. The error bars represent the standard errors of three independent experiments.

To further improve the enzymatic hydrolysis efficiency of mannan in SCG, the effects of two non-ionic surfactants, Tween 80 and PEG 6000, on the hydrolysis of aqueous ammonia-pretreated SCG were investigated (Fig. 3). The results showed that the addition of non-ionic surfactants significantly enhanced the yields of both MOS and monosaccharides, with the improvement increasing in a dose-dependent manner. When the concentration of Tween 80 and PEG 6000 reached 5 mg/mL, the MOS yields increased by 11.2 % and 34.6 %, respectively, while the monosaccharide yields improved by 51.5 % and 46.8 %, respectively. These findings indicate that Tween 80 is more effective in enhancing MOS production, whereas PEG 6000 shows a stronger effect on increasing monosaccharide yield. Therefore, from the perspective of MOS production, Tween 80 appears to be the more suitable additive during the enzymatic hydrolysis of SCG. In the presence of Tween 80, the MOS yield reached 62.3 % (119.3 mg/g raw SCG), while the monosaccharide yield was 33.9 %. These results suggest that galactomannan was almost completely degraded under these conditions, leaving cellulose and lignin as the major components of the enzymatic hydrolysis residue.

Cho et al. (2022) used NaOH and hydrogen peroxide to pretreat oil-extracted SCG and extract mannan. Subsequent enzymatic hydrolysis of the extracted mannan yielded 102 mg of MOS per gram of raw SCG. In our previous study, a two-step enzymatic hydrolysis strategy was employed, in which SCG was first hydrolyzed with MAN, followed by a second hydrolysis step using a combination of cellulase and MAN to further degrade the residual solids (Xin et al., 2024). This approach achieved a MOS yield of 122.2 mg/g raw SCG, which is comparable to the yield obtained in this study using Tween 80-assisted hydrolysis. These results indicate that the addition of Tween 80 not only enables high MOS yield but also significantly reduces the required enzyme dosage and simplifies the hydrolysis process.

To further elucidate the mechanism behind the enhancement of enzymatic hydrolysis by surfactants, the protein concentration in the hydrolysate before and after surfactant addition was measured (Fig. S2). As shown in the figure, the protein content in the hydrolysate increased significantly after the addition of surfactants. When the concentration of Tween 80 and PEG 6000 reached 5 mg/mL, the enzyme protein content increased from 0.048 mg/mL to 0.094 mg/mL and 0.058 mg/mL, respectively. These results suggest that the addition of surfactants effectively reduced the non-productive adsorption of MAN onto lignin, thereby increasing the amount of free MAN available in the hydrolysate for interaction with mannan substrates. Moreover, Tween 80 demonstrated a stronger ability than PEG 6000 in reducing the non-productive adsorption of MAN. Similarly, Huang et al. (2022) reported that Tween 80 was more effective than PEG 4000 in dissociating the irreversible adsorption of cellulase onto lignin, which aligns with the trend observed in the present study. Their findings also suggested that Tween 80 primarily disrupts enzyme–lignin interactions via hydrophobic interactions, whereas PEG-type surfactants interfere mainly through hydrogen bonding or van der Waals forces. Based on this, it can be inferred that, in the present system, the interaction between MAN and lignin in the aqueous ammonia-pretreated SCG is predominantly governed by hydrophobic forces, while interactions involving hydrogen bonding or van der Waals forces are relatively weaker. Nevertheless, the specific underlying mechanisms require further investigation in subsequent experiments.

## 4 Conclusions

This study presents a new strategy for valorizing SCG into MOS through sequential aqueous ammonia pretreatment and surfactant-assisted enzymatic hydrolysis. Under the optimized conditions (25 % aqueous ammonia pretreatment for 48 h followed by mannanase hydrolysis with 5 mg/mL Tween 80), the degradation efficiency of mannan in SCG was enhanced by approximately fivefold, and the MOS yield increased by 4.7 times, reaching 62.3 %. These findings demonstrate that the integrated application of aqueous ammonia pretreatment, MAN hydrolysis, and surfactant addition provides a promising and efficient approach for high-yield MOS production from SCG.

## CRediT authorship contribution statement

**Donglin Xin:** Writing – original draft, Investigation, Data curation, Conceptualization. **Hong Yin:** Investigation. **Zhenni Jiao:** Investigation. **Ganqiao Ran:** Supervision.

## Declaration of competing interest

The authors declare that they have no known competing financial interests or personal relationships that could have appeared to influence the work reported in this paper.

## Data Availability

Data will be made available on request.

## Appendix A Supplementary data

Supplementary material Image 1
