# Supplementary material for: Integrated alkaline pretreatment and surfactant-assisted hydrolysis for high-yield Manno-oligosaccharides production from spent coffee grounds
Source: Food Chem X. 2025 Aug 6;29:102892. doi: 10.1016/j.fochx.2025.102892 (PMC12355980; doi:10.1016/j.fochx.2025.102892)
Supplement: Supplementary file 1 — Supplementary material [file mmc1.docx]

**Supplementary materials**

**Integrated** **Alkaline Pretreatment and Surfactant-Assisted Hydrolysis for High-Yield Manno-Oligosaccharides Production from Spent Coffee Grounds**

Table S1. Chemical compositions of nonpretreated and pretreated spent coffee ground.

| Pretreatment | Cellulose (%) | Mannan (%) | Galactan (%) | Lignin  (%) | Lignin removal (%) | Cellulose recovery (%) | Mannan recovery (%) |
| --- | --- | --- | --- | --- | --- | --- | --- |
| Nonpretreatment | 12.7±0.2 | 17.7±0.2 | 8.7±0.3 | 36.6±1.4 |  |  |  |
| 50°C-24 h | 19.8±0.9 | 26.4±0.8 | 7.8±0.1 | 25.7±0.3 | 59.1 | 90.8 | 86.9 |
| 70°C-24 h | 22.1±1.4 | 31.2±0.1 | 8.3±0.3 | 22.6±1.3 | 67.0 | 92.3 | 94.1 |
| 70°C-48 h | 21.3±0.2 | 31.1±0.1 | 7.4±0.3 | 21.5±0.5 | 67.1 | 93.9 | 98.1 |

pretreatment conditions: NH_3_·H_2_O (25%), 50-70 °C, 24-48 h, S/L ratio 1:10.

Table S2. The formations of MOS from enzymatic hydrolysis of aqueous ammonia pretreated SCG by MAN for 48 h. Data are shown as the mean ± the standard error of three independent experiments.

| Pretreatment | Mannobiose (%) | Mannotriose (%) |
| --- | --- | --- |
| Raw material | 12.4±0.7 | 0.9±0.0 |
| 50°C-24 h | 32.7±1.5 | 3.0±0.1 |
| 70°C-24 h | 37.0±1.6 | 3.4±0.2 |
| 70°C-48 h | 40.3±0.2 | 3.8±0.1 |

Fig. S1. Hydrolysis of spent coffee ground pretreated by aqueous ammonia at 70℃ for 24 h by MAN (0.05, 0.1, 0.5, 1.0, and 5.0 mg/g DM) at 60 ℃ and pH 5.0 for 24 h. The error bars represent the standard errors of three independent experiments.

Fig. S2. The amount of protein during the hydrolysis of aqueous ammonia pretreated spent coffee ground by MAN (1 mg/g DM) and surfactants at 60 ℃ and pH 5.0 for 48 h. The error bars represent the standard errors of three independent experiments.
